# Investigating the Role of FGF18 in the Cultivation and Osteogenic Differentiation of Mesenchymal Stem Cells

**DOI:** 10.1371/journal.pone.0043982

**Published:** 2012-08-24

**Authors:** Eunyi Jeon, Ye-Rang Yun, Wonmo Kang, Sujin Lee, Young-Hyag Koh, Hae-Won Kim, Chang Kook Suh, Jun-Hyeog Jang

**Affiliations:** 1 Department of Biochemistry, Inha University School of Medicine, Incheon, Republic of Korea; 2 Institute of Tissue Regeneration Engineering, Dankook University, Cheonan 330–714, Republic of Korea; 3 Department of Denatal Laboratory Science and Engineering, Korea University, Seoul, Republic of Korea; 4 Department of Nanobiomedical Science and WCU Research Center, Dankook University Graduate School, Cheonan, Republic of Korea; 5 Department of Biomaterials Science, School of Dentistry, Dankook University, Cheonan, Republic of Korea; 6 Department of Physiology and Biophysics, Inha University School of Medicine, Incheon, Republic of Korea; University of Minho, Portugal

## Abstract

Fibroblast growth factor18 (FGF18) belongs to the FGF family and is a pleiotropic protein that stimulates proliferation in several tissues. Bone marrow mesenchymal stem cells (BMSCs) participate in the normal replacement of damaged cells and in disease healing processes within bone and the haematopoietic system. In this study, we constructed FGF18 and investigated its effects on rat BMSCs (rBMSCs). The proliferative effects of FGF18 on rBMSCs were examined using an MTS assay. To validate the osteogenic differentiation effects of FGF18, ALP and mineralization activity were examined as well as osteogenic differentiation-related gene levels. FGF18 significantly enhanced rBMSCs proliferation (*p*<0.001) and induced the osteogenic differentiation by elevating ALP and mineralization activity of rBMSCs (*p*<0.001). Furthermore, these osteogenic differentiation effects of FGF18 were confirmed via increasing the mRNA levels of collagen type I (Col I), bone morphogenetic protein 4 (BMP4), and Runt-related transcription factor 2 (Runx2) at 3 and 7 days. These results suggest that FGF18 could be used to improve bone repair and regeneration.

## Introduction

Fibroblast growth factor18 (FGF18) belongs to the FGF family and is most homologous to FGF-8 and FGF-17 among the FGF family [1]. FGF18 is first reported in 1998 [2]. FGF18 is expressed primarily in the adult lungs and kidneys as well as in several discrete regions at embryonic days 14.5 and 19.5 [1]. FGF18 is known as a pleiotropic protein that stimulates proliferation in several tissues. In addition, FGF18 has been extensively studied as a factor of endochondral bone development (endochondral ossification) via the regulation of chondrogenesis and osteogenesis [3]–[6]. FGF18 contributes to the control of mature chondrocytes and their progenitors and is a positive regulator of osteogenesis. FGF2 is another member of FGF family that promotes osteoblast differentiation in MSCs [7], [8]. Notably, Shimoaka et al. reported that FGF18 may compensate for the roles of FGF2 in a physiological and pathological condition and might be clinically applicable as a potent osteogenic agent [9].

Mesenchymal stem cells (MSCs) are multipotent stem cells that can differentiate into a variety of cell types [10], including: osteoblasts, chondrocytes and adipocytes. MSCs found in many adult tissues are an attractive stem cell source for the regeneration of damaged tissues in clinical applications because they are characterized as undifferentiated cells, able to self-renew with a high proliferative capacity, and possess a mesodermal differentiation potential [11]. Therefore, the osteogenic potential of MSCs is speculated to improve for optimal bone regeneration [12]–[14]. The osteogenic differentiation of MSCs is identified by the expression of genes such as Runt-related transcription factor 2 (Runx2), alkaline phosphatase (ALP) and collagen type I (Col I) followed by extracellular matrix mineralization [15], [16]. Among MSCs, bone marrow mesenchymal stem cells (BMSCs) are important progenitors and can self-renew and differentiate into multiple functional cells [17]. In addition, BMSCs participate in the normal replacement of damaged cells and in disease healing processes within bone and the haematopoietic system [18], [19]. In particular, BMSCs are a major source of osteoblasts for bone remodeling and repair in postnatal animals [20]. BMSCs can circulate to bone surfaces, and proliferate and differentiate into functional cells. Thus, BMSCs are widely used in many studies.

In this study, we constructed recombinant FGF18 and investigated the effect of FGF18 on rat BMSCs (rBMSCs). We investigated the mitogenic activity and the osteogenic differentiation activity of FGF18 on rBMSCs.

## Results

### Expression and purification of recombinant FGF18 in *E. coli*

*E. coli* TOP10 cells were grown overnight in LB-Amp medium at 37°C. When the culture reached an A_600_ value of 0.5, induction was initiated with 0.02% (w/v) L-arabinose as inducer. After 3 h, bacteria were pelleted by centrifugation, lysed, and sonicated. Crude protein from cell extract was purified by binding His_6_ tag (located at the N-terminal end of FGF18) to the nickel-nitrilotriacetic acid resin column. The purity of the recombinant protein was examined under denaturing condition by coomassie blue staining of 12% SDS-PAGE gel.

**Figure 1:**
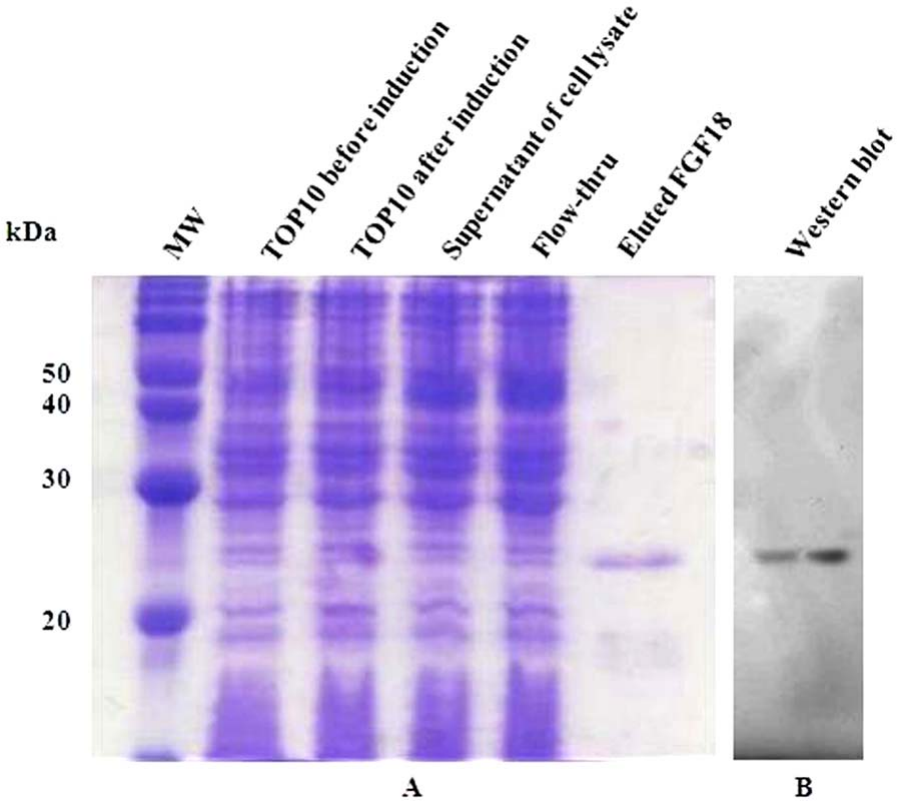
Expression of purified recombinant FGF18 by SDS-PAGE. A) Proteins were subjected 12% (w/v) SDS-PAGE gel under reducing condition and visualized by coomassie blue staining. B) This western blot shows a protein band at approximately 25 kDa.

Upon induction with L-arabinose, *E. coli* TOP10 produced a protein of molecular weight (MW) *ca*. 25 kDa (as estimated by SDS-PAGE), which was the size expected for a fusion protein consisting of FGF18 and the amino-terminal His_6_ tag. The expression of recombinant FGF18 was confirmed by western blotting using a peroxidase conjugate of a monoclonal anti-polyhistidine antibody (Fig. 1).

### Enhanced proliferative activity of FGF18 on rBMSCs

**Figure 2:**
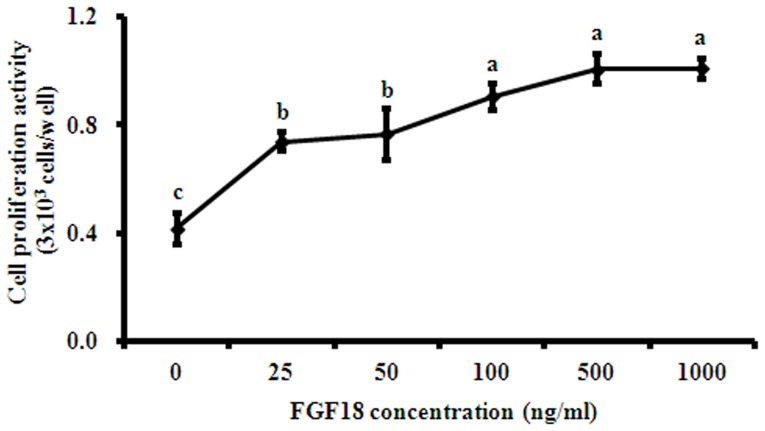
Cell proliferative effect of FGF18 on rBMSCs at 5 days. Cells were seeded at a density of 2.5×10^3^ cells/well on plates coated with FGF18 and incubated overnight at 4°C. Formazan absorbance was used as a measure of cell proliferation. Cell proliferation activity was expressed as cell numbers (3×103 cells/well) by MTS assay and represent the mean±SD. ^a–c^Different letters show statistically significance difference among groups (*p*<0.001).

The effect of FGF18 on the proliferative activity of rBMSCs was examined using a MTS assay. As shown in Fig. 2, the proliferative activity of FGF18 was dose-dependently greater than that of non-treated control (*p*<0.001). Based on these proliferative results, 500 ng/ml FGF18 was used in subsequent experiments.

### Osteogenic differentiation activity of FGF18 on rBMSCs

**Figure 3:**
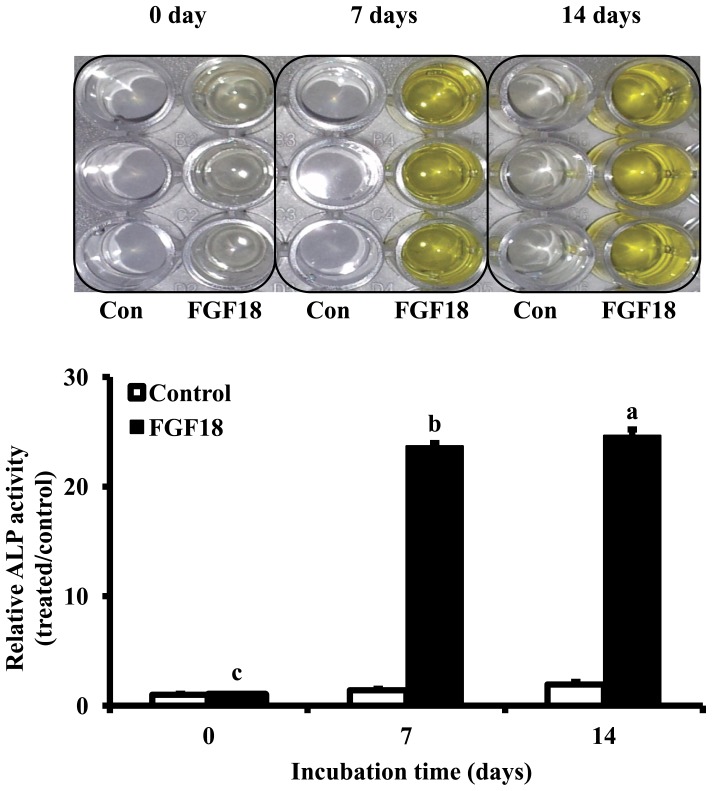
ALP activity of FGF18 (500 ng/ml) on rBMSCs at 0, 7 and 14 days. Cells were seeded at a density of 2.5×10^3^ cells/well on plates coated with or without FGF18 and incubated overnight at 4°C. Non-treated cells were used as a control. ALP activity was normalized versus control and represent the mean±SD. ^a–c^Different letters show statistically significance difference among groups (*p*<0.001).

**Figure 4:**
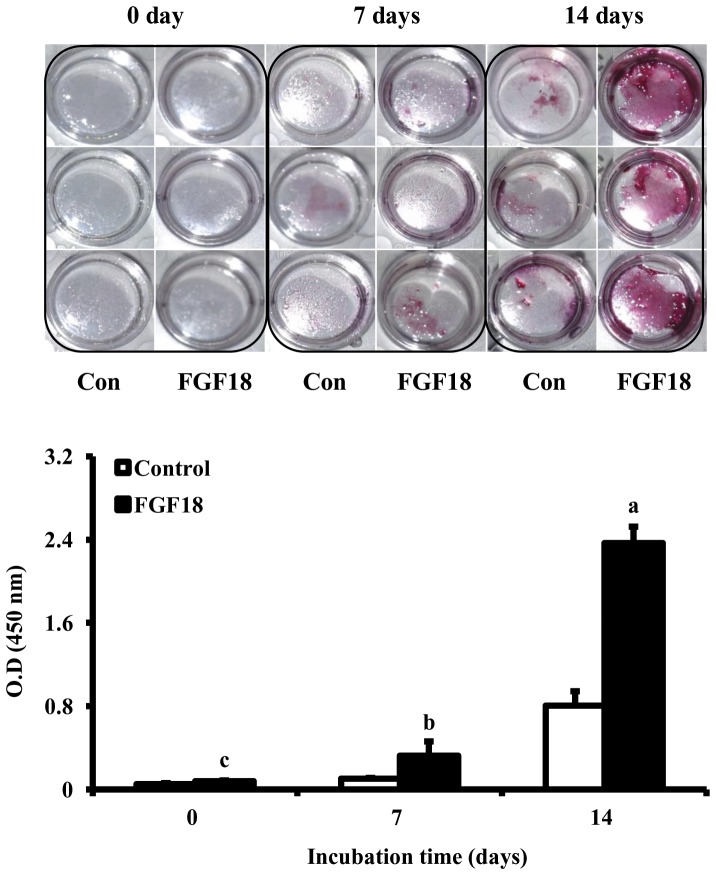
Mineralization activity of FGF18 (500 ng/ml) on rBMSCs at 0, 7 and 14 days. Cells were seeded at a density of 1×10^4^ cells/well on plates coated with or without FGF18 and incubated overnight at 4°C. Non-treated cells were used as a control. Mineralization activity was expressed as absorbance and represent the mean±SD. ^a–c^Different letters show statistically significance difference among groups (*p*<0.001).

The effect of FGF18 on the osteogenic differentiation activity of rBMSCs was determined using ALP activity. FGF18 significantly elevated ALP activities of rBMSCs compared to the non-treated control 7 and 14 days (*p*<0.001, Fig. 3). These osteogenic differentiation activities of FGF18 were confirmed in mineralization activity. To measure mineralization activity, alizarin red s staining images were captured and quantitative assay was measured. Similarly, FGF18 significantly increased mineralization activity of rBMSCs 7 and 14 days compared to the non-treated control (*p*<0.001, Fig. 4). These results indicate that FGF18 could induce the osteogenic differentiation of rBMSCs by elevating ALP and mineralization activity.

### Osteogenic differentiation gene expression by real-time PCR

**Figure 5:**
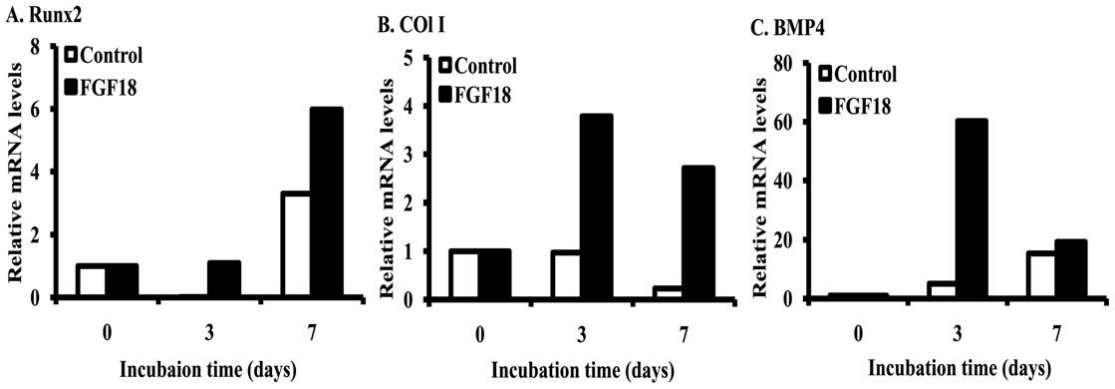
The osteogenic differentiation of FGF18 (500 ng/ml) on rBMSCs at 3 and 7 days. A) Runx2, B) Col I, C) BMP4.

We also examined the effects of FGF18 on the osteogenic differentiation of rBMSCs by examining the expression of Col I, BMP4, and Runx2 mRNA. FGF18 was found to increase the expression of all three of these osteogenic differentiation markers (Fig. 5).

## Discussion

FGF18 has been demonstrated to play a pivotal role in proliferation and differentiation in MSCs similar to FGF2. For instance, recombinant murine FGF18 (rMuFGF18) stimulated the proliferation in the fibroblast cell line *in vitro*, and induced the proliferation in epithelial and mesenchymal origin *in vivo* [21]. In addition, rhFGF18 has been shown to participate in limb bud mesenchymal cells by suppressing the proliferation and promoting differentiation in cartilage matrix [22]. Notably, rhFGF18 has been also involved in osteogenic differentiation by inducing the osteoblast gene expression on murine MSCs including BMC10 murine mesencymal stem cell and murine C3H10T1/2 stem cells [23]. Taken together, FGF18 may play a crucial role in proliferation and differentiation on MSCs.

In this study, recombinant FGF18 was expressed in *E. coli*, and its effects of on rBMSCs their mitogenic and osteogenic differentiation activities are described. Furthermore, FGF18 was found to have a dose-dependent effect on the viability of rBMSCs (Fig. 2). Thus, we speculate that FGF18 has a mitogenic effect on rBMSCs, which is consistent with the finding of previous studies. In addition, FGF18 was found to stimulate the proliferation in several organs, such as, the liver and the small intestine as a pleiotropic GF [21]. FGF18 has been previously reported to activate the proliferations in different cell types [24]–[26]. Interestingly, Shimoaka et al. suggested that the mitogenic activity of FGF18 is more specific to bone and cartilage cells than FGF2 [9].

FGF18 is known to participate in osteogenesis as well as chondrogenesis [3]–[6]. Osteogenic differentiation is generally accompanied by osteogenic differentiation gene markers, and *in vitro* mineralization. Thus, we examined ALP activity and alizarin red s staining of rBMSCs treated with FGF18 to validate the osteogenic differentiation activity of FGF18, and found that ALP activity was significantly increased by FGF18 (*p*<0.001, Fig. 3). Furthermore, these osteogenic differentiation activities of FGF18 were confirmed in mineralization activity. Quantitative assay showed that the osteogenic differentiation of rBMSCs was significantly increased compared with untreated control at 7 and 14 days after FGF18 treatment (*p*<0.001, Fig. 4). Osteogenic differentiation by FGF18 has been previously reported in murine MSCs [23]. FGF18 is an essential positive autocrine regulator of the osteogenic differentiation in murine MSCs, and the osteogenic differentiation induced by FGF18 in MSCs was found to be triggered by FGFR1/FGFR2-mediated ERK1/2-MAPKs and PI3K signaling, which indicates that FGF18 is required for osteogenesis.

To confirm the osteogenic differentiation activity of FGF18, the mRNA levels of osteogenic differentiation gene markers were measured by real-time PCR. Particularly, the expression of Runx2, Col I, and BMP4 were measured. These genes are involved in the early osteogenic differentiation. The present study shows that FGF18 stimulated the up-regulation of genes associated with the osteogenic differentiation including Runx2, Col I, and BMP4 by FGF18 on rBMSCs (Fig. 5). In particular, the gene expressions of Col I, and BMP4 were dramatically up-regulated by FGF18 at 3 days. Runx2 is known to act as a key transcription factor during osteoblast differentiation [27]. For instance, Runx2 is involved in the differentiation of human MSCs (hMSCs) into osteogenesis in early stage [28]. These results are consistent with our present study. Col I is well-known as component of extracellular matrix (ECM) and the most abundant collagen. In addition, Col I is found in scar tissue, tendons, the endomysium of myofibrils, and the organic parts of bone. During osteoblast differentiation, Col I is gradually down-regulated [29]. Interestingly, the gene expression of Col I by FGF18 was increased versus untreated control. BMP4 is a member of the bone morphogenetic protein family which is part of the transforming growth factor-beta superfamily (TGF-β). Furthermore, BMP4 plays an important role in osteogenic differentiation [30] as well as other genes, and stimulates the differentiation of overlying ectodermal tissue [31]. However, FGF18 did not stimulate the late osteogenic differentiation genes such as osteocalcin (OCN) (data not shown). Taken together, FGF18 participates in the early osterogenic differentiation of rBMSCs.

In our study, we could not investigate the function of FGF18 *in vivo*. However, earlier study has been revealed that FGF18 inhibits chondrogenesis through FGF receptor 3 (FGFR3). While, Fgf*18* null mice have been shown to down-regulate the preosteogenic and osteogenic markers through another FGFR. These results suggested that FGF18 may block chondrogenesis and promote osteogenesis *in vivo* like *in vitro* [6]. In other study, application of FGF18 with collagen sponge rescued the impaired healing of tibial defects in Fgf*18* null mice [5]. Based on our results and these results *in vivo*, FGF18 is expected as a useful FGFs for application in bone tissue engineering.

In conclusion, the present study shows FGF18 acts as a strong mitogenic factor, and that it induces the osteogenic differentiation on rBMSCs, which is expected to lead to improve bone repair and regeneration.

## Materials and Methods

### Construction and purification of recombinant FGF18

PCR primers were designed to recognize human FGF18 as follows; FGF18 forward primer, 5′-TGGTACCTGTACCAGCTCTACAGC-3′, FGF18 reverse primer, 5′-TGGTACCA CTAGGCAGGGTGTGTGG-3′. PCR was performed in 30 µl reaction mixes containing 50 mM KCl, 10 mM Tris-HCl (pH 8.3), 1.5 mM MgCl_2_, 100 µg/ml gelatin, 0.2 mM dNTPs, 1.25 units of Taq polymerase (iNtRON, Seoul, Korea), and 50 pmol each of the forward and reverse primers. PCR was conducted over 30 cycles 1 min at 55°C (annealing), 1 min at 72°C (extension), and 1 min at 94°C (denaturation). Amplified products were digested with *KpnI*, ligated into the *KpnI* sites of pBAD/HisA vector (Invitrogen, Carlsbad, CA, USA), which gave rise to the pBAD/HisA-FGF18 construct.

To express recombinant FGF18, *E. coli* TOP10 cells were grown overnight in LB-Amp medium at 37°C. When the cultures reached an A_600_ of 0.6, induction was initiated with 0.02% (w/v) L-arabinose. Three hours later, bacteria were pelleted by centrifugation at 6,000 x g for 10 min, lysed, and sonicated. A soluble extract was prepared by centrifugation at 14,000 x g for 30 min in a refrigerated centrifuge and the supernatant so obtained was transferred to a fresh tube.

The crude protein obtained from the sonicated bacterial supernatant was purified via the binding of the hexahistidine tag (located at the amino-terminal end of FGF18) to a nickel-nitrilotriacetic acid resin column, according to the manufacturer's instructions (Invitrogen, Carlsbad, CA, USA). The degree of purification of the recombinant protein was determined under denaturing conditions by coomassie blue staining of 12% (v/v) SDS-PAGE gel.

Western blots were performed using a peroxidase conjugate of a monoclonal anti-polyhistidine antibody (sc-8036, Santa Cruz Biotechnology, Santa Cruz, CA, USA) to confirm the expression of the recombinant FGF18. The molecular sizes of the immunodetected protein were verified by comparison to the migration of pre-stained protein markers (Elpis Biotech, Daejeon, Korea) eletrophoresed in parallel lanes.

### Cell culture

rBMSCs were cultured in α-minimum essential medium (Invitrogen, Carlsbad, CA, USA) containing 10% heat-inactivated fetal calf serum (Invitrogen, Carlsbad, CA, USA), 100 units/ml penicillin G sodium, 100 µg/ml streptomycin sulfate, and 0.25 µg/ml amphotericin B (Invitrogen, Carlsbad, CA, USA) in a 5% CO_2_ atmosphere at 37°C. Confluent cells were detached with 0.25% trypsin-EDTA for 5 min, and aliquots were subcultured. rBMSCs maintained for 3 passages were used for further cell proliferation and differentiation study.

### Cell proliferation assay

Proliferation was assessed using an MTS (3-(4,5-dimethylthiazol-2-yl)-5-(3-carboxy-methoxyphenyl)-2-(4-sulfophenyl)-2H-tetrazolium) tetrazolium viability assay (Promega, Madison, WI, USA), as described by the manufacturer. This assay measures the conversion of a methyl tetrazolium sulfate (MTS) to aqueous soluble formazan product that absorbs at 490 nm. Two hours after adding MTS (40 µl) the plates were read at 490 nm.

### Cell differentiation assay

To assess ALP activities, rBMSCs were incubated at a density of 2.5×10^3^ cells/well for 0, 7 and 14 days in differentiation-inducing media without or with FGF18. Differentiation-inducing media contained 100 µmol/l/ml ascorbic acid, 2 mmol/l β-glycerophosphate, and 10 nmol/l dexamethasone. Then, cells were rinsed with phosphate-buffered saline (PBS), and lysed with 1.5 M Tris-HCl (pH 10.2) containing 1 mM ZnCl_2_, 1 mM MgCl_2_ and 1% Triton X-100 at 4°C for 10 min. Following clarification by centrifugation, cell lysates were assayed for ALP activity using an Alkaline Phosphate Assay Kit (Sigma, St. Louis, MO, USA). *p*-Nitrophenol was produced in the presence of alkaline phosphatase and its absorbance was determined spectrophotometrically at 405 nm using a microplate reader (BioRad Laboratories, CA, USA). ALP activities were normalized versus control.

### Alizarin red s staining

To assess the osteogenic differentiation inducing efficacy of FGF18, rBMSCs were cultured at a 1×10^4^ cells/well for 0, 7, and 14 days in differentiation media, rinsed with PBS, fixed in 95% methanol for 30 min, and stained in alizarin red s solution overnight. Stained cells were then photographed at 0, 7 and 14 days. Next, stained cells were followed by a quantitative destaining procedure using 10% (w/v) cetylpyridinium chloride in 10 mmol sodium phosphate (pH 7.0) for 30 min at room temperature. The alizarin red s concentration was determined by absorbance measured at 450 nm on a spectrophotometer.

### RNA extraction and cDNA synthesis

Total RNA was extracted using an Easy-spin RNA Extraction kit (iNtRON, Seoul, Korea). RNA purity was assessed by absorption at 260 and 280 nm (A_260_/A_280_ ratio values of 1.9–2.1 were considered acceptable) and by ethidium bromide staining of 18 S and 28 S RNA on gel electrophoresis. RNA concentrations were determined from A_260_ values. Two micrograms of total RNA were reverse-transcribed in a 20 µl reaction mixture containing 50 units of SuperScript II reverse transcriptase (Invitrogen, Carlsbad, CA, USA), 5 µM DTT, 40 units of RNaseOUT recombinant ribonuclease inhibitor 0.5 µM of random hexanucleotide primers, and 500 µM of dNTP mixture. Reverse transcription was carried out at 50°C for 60 min. The reaction mixture was subsequently heated at 70°C for 15 min to terminate the reaction. The cDNA so obtained was stored at −20°C.

### Quantitative real-time PCR analysis

The over-expressions of the genes obtained were confirmed by quantitative real-time PCR. All real-time PCR analyses were performed on an ABI Step One real-time PCR system. Each reaction was performed in a 20 µl reaction mixture containing 0.1 µM of each primer, 10 µl of 2xSYBR Green PCR master mix (Applied Biosystem, including AmpliTaq Gold DNA polymerase in buffer, a dNTP mix, SYBR Green I dye, Rox dye, and 10 mM MgCl_2_), and 1 µl of template cDNA. PCR was conducted by activation the enzyme at 94°C for 10 min followed by 40 cycles of denaturation at 94°C for 15 s, annealing at 60°C for 1 min, and extension at 60°C for 1 min. The *C_T_* (cycle threshold) value for each gene was determined using the automated threshold analysis function in the ABI instrument and normalized with respect to *C_T_*_(GAPDH)_ to obtain d*C_T_* ( = *C_T_*_(GAPDH)_ - *C_T_*_(specific gene)_). A difference of *n* between two *C_T_* or d*C_T_* values indicated a 2*^n^*-fold difference in the target sequence between the two cDNA samples being compared. The primers used for quantitative PCR are shown in Table 1.

**Table 1:** Sequences of primers used for real-time PCR.

Genes	Forward primer	Reverse primer
*GAPDH*	TGGAAGGACTCATGACCACA	TTCAGCTCAGGGATGACCTT
*Runx2*	CGCCCCTCCCTGAACTCT	TGCCTGCCTGGGATCTGTA
*Col I*	CTGGCAAGAACGGAGATGAT	TTAGGACCAGCAGGACCAGT
*BMP4*	GAACAGGGCTTCCACCGTATAAAC	TGTCCAGTAGTCGTGTGATGAGG

### Statistical analysis

Data were expressed in mean±SD. Statistical analysis were performed using SAS program among groups. Differences between two groups were tested by Student t-test with unpaired data set. In all statistical analyses, a *p* value below 0.05 was considered as significant.
