# Correction: Investigating the Role of FGF18 in the Cultivation and Osteogenic Differentiation of Mesenchymal Stem Cells

**DOI:** 10.1371/annotation/77eec9d5-379f-4895-8b85-308ee29facf4

**Published:** 2012-10-25

**Authors:** Eunyi Jeon, Ye-Rang Yun, Wonmo Kang, Sujin Lee, Young-Hyag Koh, Hae-Won Kim, Chang Kook Suh, Jun-Hyeog Jang

The fifth author's name was spelled incorrectly. The correct name is: Young-Hag Koh.

